# Influence of flow on phosphorus-dynamics and particle size in agricultural drainage ditch sediments

**DOI:** 10.1371/journal.pone.0227489

**Published:** 2020-01-13

**Authors:** Jay Capasso, Jehangir H. Bhadha, Allan Bacon, Lilit Vardanyan, Raju Khatiwada, Julio Pachon, Mark Clark, Timothy Lang

**Affiliations:** 1 UF IFAS Columbia County Extension, University of Florida, Lake City, Florida, United States of America; 2 Everglades Research and Education Center, Soil and Water Sciences Department, University of Florida, Belle Glade, Florida, United States of America; 3 Soil and Water Sciences Department, University of Florida, Gainesville, Florida, United States of America; Central South University, CHINA

## Abstract

Particle size is one factor affecting phosphorus (P) dynamics in soils and sediments. This study investigated how flow facilitated by hydraulic pumps and aquatic vegetation species water lettuce (*Pistia stratiotes*) and water hyacinth (*Eichhornia crassipes*) affected particle size and P-dynamics in organic sediments in agricultural drainage ditches. Sediments with finer particle size (>0.002 mm) were hypothesized to contain greater total P (TP) and less labile P than sediments with coarser particle size. Particle size was determined using a LS 13 320 Laser Diffraction Particle Size Analyzer. Sediments were tested for pH, TP, and organic matter. Fractions of P were determined using a sequential fractionation experiment and ^31^P Nuclear Magnetic Resonance (NMR) Spectroscopy. Larger average particle size and lower average total P concentrations were found in the inflows of the field ditches compared to the outflows. Presence of flow and aquatic vegetation did not have a significant impact on particle size, TP, or labile P fractions. Median (p = 0.10) particle size was not significantly correlated to TP. Overall, there was an average trend of coarser particle size and lower P concentrations in the inflow compared to the outflow. The presence of inorganic limerock could have affected results due to increased P adsorption capacity and larger average particle size compared to the organic fraction of the sediment.

## Introduction

Phosphorus (P) from non-point sources such as agriculture increase eutrophication, potentially causing algal blooms, fish kills, and subsequent environmental and economic harm [1]. The Everglades Agricultural Area (EAA) sits upstream from the Florida Everglades, a traditionally P limited environment. Additions of P to the Everglades have been found to cause eutrophication and changes in vegetation communities, such as when the naturally dominant sawgrass (*Cladium jamaicense*) is replaced by cattail (*Typha)* [2,3]. There is also a need to reduce P loads in water flowing south towards the Florida Everglades to achieve the 0.010 mg L ^-1^ P concentration required by law under the Everglades Forever Act [4]. A better understanding of transport and bioavailability of P is needed to reduce the export of P into surface waters.

The EAA is approximately 280,000 hectares of the original northern Everglades, where anaerobic conditions over a 4,400 year period facilitated the production of organic soils due to reduced decomposition rates of aquatic vegetation (primarily sawgrass) [5]. Draining the area in the early 20^th^ century resulted in aerobic soil conditions that facilitated microbial oxidation of organic matter causing soil subsidence [6]. Soils are classified as histosols with the suborder sapric, meaning that organic matter texture is well decomposed [5]. Traditionally, soils were acidic, and according to reports from the late 1980s, organic matter content typically ranges between 80 to 90% [7,8]. However, due to soil subsidence and the underlying limerock, pH has risen over time, and organic matter content is more commonly found in the 70–80% range. In canal sediments, commonly dredged, the average organic matter content is approximately 40% [9].

In the Everglades region, there are over 2,500 km of agricultural drainage ditches, canals, and levees that support drainage of agricultural land and municipalities. About 40% to 60% of P loads exported from EAA farms is particulate P, primarily originating from in-stream biological growth of algae, plankton, and aquatic vegetation in agricultural drainage ditches and canals [9,10]. One method farmers use to reduce particulate P runoff is through the physical and chemical management of floating aquatic vegetation. Floating aquatic vegetation can remove soluble P from the water column through uptake of nutrients and facilitate sedimentation of suspended particles as water flow encounters resistance from vegetation [11,12]. Vegetation is also expected to influence P-dynamics of sediment through altering redox potentials by physically covering the water column preventing oxygen exchange, and by senescing, contributing to the mineralization of P and the accumulation of flocculent organic ditch sediment.

Particle size is one factor affecting P concentration and bioavailability in soils and sediments [13]. Although the role of particle size in affecting P-dynamics is not completely understood, it is believed to influence P adsorption, desorption, and diffusion, as well as the biological processes of mineralization and immobilization [14]. Phosphorus solubility has been found in isotherm studies to be directly related to soil texture [15,16]. In these studies, P has been found to be more soluble in coarser particle sizes and less available in finer particle sizes [17,18]. Huffman et al. (1996) found particle size to affect the transformation of labile P more than additions of crop residues and nutrients combined [19]. In a mollisol, particle size explained 90% of the variability in TP [19,20]. In EAA sediments, the P cycle is influenced by various factors other than particle size, such as organic matter content, pH, and redox potential. Hydrous iron (Fe) and aluminum (Al) oxyhydroxide (FeO(OH), AlO(OH)) and calcium carbonate (CaCO_3_) content are expected to have important roles in regulating P sorption [21,22]. Phosphorus fractionation procedures have been developed to measure bioavailability of P and the distribution of P in different chemical fractions. However, there has been little investigation on whether the particle size of organic matter affects P solubility and TP.

This study investigated how flow, facilitated by hydraulic pumps, and the introduction of floating aquatic vegetation affected sediment texture and P dynamics in agricultural drainage ditches in the EAA. Introduced flow is hypothesized to cause coarser particles to settle near the inflow and finer textured particles to settle near the outflow of the ditch. Floating aquatic vegetation is hypothesized to further facilitate sedimentation of particles. Objectives of this study were to determine (i) if introduced flow causes coarser textured sediments to accumulate near the inflow location compared to the outflow and (ii) whether any subsequent changes occur in P-dynamics as a result of flow.

## Materials and methods

### 2.1 Experimental design

In order to determine the effectiveness of our treatment method, four treatment ditches were equipped with hydraulic pumps that provided flow to the ditches (ditches 1–4). The floating aquatic vegetation water lettuce (*Pistia stratiotes*) and water hyacinth (*Eichhornia crassipes*) were introduced and established in treatment ditches 2 and 3, respectively, in March 2017. Two control ditches (ditches 5 and 6) were kept without hydraulic pumps or introduced aquatic vegetation. All six ditches were dredged by farm management in January 2017 with automotive machinery to move sediment from the bottom of the ditches to adjacent sugarcane fields.

### 2.2 Methods

Sediments were collected from inflow and outflow of six agricultural field ditches located in sugarcane fields in the EAA. Samples were collected in 7.5 cm diameter Plexiglas core tubes and transported to the Soil, Water and Nutrient Management Laboratory at the Everglades Research and Education Center where they were stored at 4°C for 24 h to allow suspended particles to settle. After 24 h, water was siphoned out of the sampling tubes and the upper 5 cm of sediment were collected and stored at 4°C in plastic containers. Sediments were collected from the same locations in the field ditches on three different dates over a one year period (11.03.2016, 05.09.2017, and 11.08.2017). Sediment depths were measured using a meter stick and taking measurements every 40 m along each of the six ditches. Two samples were collected for ^31^ P Nuclear Magnetic Resonance (P-31 NMR) spectroscopy from inflow and outflow of treatment ditch 3 in July 2017.

Sediments were tested for TP, pH, organic matter, particle size, sequential P fractionation modified from [23], and ^31^P NMR spectroscopy. Total P was determined using EPA method 365.4 by digesting sample with sulfuric acid (H_2_SO_4_), potassium sulfate (K_2_SO_4_), and mercuric sulfate (HgSO_4_) and analyzed using an (AutoAnalyzer 3, Seal Analytical Inc. Mequon, Wisconsin). An Accumet Ab250 pH meter and a 1:10 sediment/water extract was used to determine pH. Organic matter content was calculated using loss on ignition (LOI) at 550°C for approximately 4 h. Particle size was determined using LS 13 320 laser diffraction particle size analyzer (Beckman Coulter Inc. Brea, California). A modified Hedley (1982) method [24] was used to determine soluble P, fulvic/humic P, iron and aluminum (Fe/Al) P, calcium and magnesium (Ca/Mg) P, and residual P. Phosphorus-31 NMR spectroscopy was conducted on sediments collected on 7/19/2017 from inflow and outflow.

### 2.3 Laser diffraction particle size analysis

To determine particle size, sediments were transported to the University of Florida’s Environmental Pedology and Land Use Laboratory. Prior to analyses, samples were homogenized in their containers by shaking vigorously by hand. Next, an approximately equal volume of each sample was place in a 50 mL centrifuge tube, and 5 mL of 5% sodium-hexametaphosphate (Na_6_O_18_P_6_) was added. The slurry was vortexed for 10 s and then another 5 mL of Na_6_O_18_P_6_ was added to rinse solids off centrifuge tube walls. After sitting overnight, the slurry was introduced to a Beckman Coulter LS-13320 multi-wave particle size analyzer through an aqueous liquid module sample introduction system connected to deionized water (DI). We used a combination of pouring and rinsing with a DI squirt bottle to ensure all particles were transferred into the aqueous liquid module. Across all particle size analyses, the obscuration (an instrument parameter indicating signal to noise ratio) ranged from 3 to 15% and averaged 9%. Samples were analyzed for a total of 60 s at a pump speed of 7.2 L min^-1^. Particle size distributions were calculated following the Mie theory with the imaginary portion of the sample refractive index set to 0.2, the real portion of the sample refractive index set to 1.53, and the refractive indices of DI set to 1.332 for the 450, 600, 750, and 900 nm wavelengths [25,26].

### 2.4 Modified Hedley (1982) experiment

A P fractionation experiment was conducted using a modified Hedley (1982) method from [24]. A 1–2 g sample of air-dry sediment was sequentially extracted using 1 M potassium chloride (KCl), 0.1 M sodium hyrdroxide (NaOH), 0.5 M hydrocloric acid (HCl), and 6.0 M HCl. Samples were subsequently mechanically shaken, centrifuged at 6000 rpm for 10 min, filtrated, and analyzed using colormetric methods or Inductively Coupled Plasma Optical Emission Spectrometry (ICP). The KCl-Pi (soluble inorganic P) was determined by adding 20 mL of 1.0 M KCl and shaking for 2 h prior to centrifugation, filtration, and analysis by Genesys 30 visible spectrophotometer at 880nm [27].

Then 20 mL of 0.1 M NaOH was added and shaken with the sample for 17 h. The extractant was subsequently centrifuged, and filtrated. For NaOH TPi (inorganic Fe and Al bound P) the extractant was filtered using a 60 mL syringes and 0.45 μm filter before analysis by Genesys 30 visible spectrophotometer at 880 nm [27]. For NaOH TP, 5 mL of the extractant was pipetted into digestion vials with a 1.5 mL 11 N H_2_SO_4_ and 0.3 g of potassium persulphate (K_2_O_8_S_2_). Samples were digested at 150°C for 2 h until liquid evaporated, then samples were digested at 350°C for 4 h until colorless. Samples were then diluted with 10 mL of DI water, poured into scintillation vials, and stored at 4°C until analysis by ICP.

Finally, 20 mL of 0.5 M HCl was added to the sample and shaken for 24 h before centrifuging and filtering. Samples were stored at 4°C and analyzed by Genesys 30 visible spectrophotometer for (Ca/Mg P) at 880 nm [27]. Residual P was calculated by drying sediment at 70°C and subsequently ashing in a furnace at 550°C. Samples were transferred to digestion tubes with 20 mL of 6 M HCl. In a digestion block, samples were digested at 120°C until dry. Deionized water (2mL) and 2.25 ml of 6 M HCl were added and returned to digestion block at 120°C for 3 min. Samples were then filtered through Whatman # 41 filter paper and contents were transferred into 50 mL volumetric flasks and diluted to 50 mL volume. Samples were analyzed using ICP.

### 2.5 P-31 NMR spectroscopy

For ^31^P NMR analyses, samples were transported to the Wetland Biochemistry Laboratory in Gainesville, Florida. Three replicates from each sample were homogenized together through mixing. Moisture content was determined by subsampling from two homogenized wet samples. Each sample was subsampled three times. Moisture content was determined by weighing approximately 10 g of each wet subsample and placing them in the oven at 70°C for 48 h. Mechanically homogenized (removing visible roots and plant material) inflow and outflow samples with three replicates were placed in the oven at 35°C for ^31^P NMR analyses. Dried samples were transferred to plastic scintillation vials and ground for 10 min with an electric grinder. To determine NaOH extractable P (NaOH-TP) sediment samples were extracted with 0.25 M NaOH and 0.05 M disodium ethylenediaminetetraacetic acid (Na_2_EDTA) extraction solution (soil to solution ratio = 1:20; 16 h extraction time). Soil suspensions were centrifuged at 10,000 rpm, and supernatant solutions were digested and analyzed for P using an UV-1800 spectrophotometer at 880 nm [27].

For ^31^P NMR analysis, 20 mL centrifuged extracted alkali solutions were amended with 1 mL of methylene diphosphonic acid (MDP) (as an internal standard) and were frozen, freeze-dried, and lyophilized into powder. Lyophilized powder (300 mg) was re-dissolved in 2.7 mL resuspension solution (1 M NaOH and 0.1 M Na_2_EDTA) + 0.3 mL D_2_O [28]. After vortexing and centrifuging, samples were transferred to 5 mm NMR tubes and analyzed using a Bruker Avance-III-600 spectrometer with a 5-mm BBO-Z probe. This instrument is located at the McKnight Brain Institute of the University of Florida. The NMR parameters used for analysis were: (1) pulse program for acquisition: zgig; (2) time domain size: 19434; (3) acquisition time: 0.4; (4) pre scan delay: 8.5; (5) relaxation delay: 2.0; (6) number of dummy scans: 64; (7) t number of scans: 256; (8) duration: 24 h. Signal areas were calculated by integration and P concentrations calculated from the integral value of the MDP internal standard at 17.63 mg L ^-1^ [29,30].

### 2.6 Water flow rates and total phosphorus

Flow was provided to the four treatment ditches by hydraulic pumps (TRU-FLO Corporation. Belle Glade Florida). Pumps were inserted into the culvert that connected the treatment ditches to the main canal, pushing water from the main canal into treatment ditches. Hydraulic pumps were set to operate on a 10:10 min on:off schedule from 8:00 am to 6:00 pm throughout the experiment. Flow rates were measured at the inflow of the treatment ditches using a Marsh-McBirney, Inc. Flo-Mate 2000. Average mid ditch velocity rates were calculated by multiplying the average width and depth (m^2^) by the average mid canal flow rate (m^3^ s ^-1^). Pumps were set at the same voltage to insure each pump provided a similar flow rate to each of the four treatment ditches. No hydraulic pumps were introduced in the control ditches, and flow rates were not measured because of minimal expected flow due to flat topography. Flow in the control ditches was reliant on water depth in the main canal and ditches. Throughout the experiment, water samples were collected from the inflow and outflow locations of ditches every two weeks. Samples were preserved in H_2_SO_4_ before being digested then analyzed by a continuous flow analyzer (Auto Analyzer 3, Seal analytical) for TP using EPA method 365.1.

### 2.7 Aquatic vegetation

Prior to the start of the experiment, the submerged aquatic vegetation muskgrass (*Chara*), southern naiad (*Najas guadalupensis*), and coontail (*Ceratophyllum demersum*) were introduced into each of the four treatment ditches but did not become established. Floating aquatic vegetation water hyacinth and water lettuce were introduced and established in treatment ditches 2 and 3, respectively. Vegetation was collected from nearby canals and agricultural drainage ditches in the EAA. No aquatic vegetation was introduced in control ditches 5 and 6. For detailed information regarding aquatic vegetation characterization and its effect on P loads within the EAA refer to [31].

### 2.8 Statistical analysis

One tailed t-tests assuming equal variance were conducted to determine differences between inflow and outflow locations of each ditch for organic matter, pH, mean, and median particle size using Microsoft Excel. In addition, data were transformed into differences in values between inflow and outflow locations. Model assumptions of normality and homogeneity of variances were checked through the visual inspection of model residuals. A linear mixed model was performed using SAS 9.4 statistical software. The model accounted for the ditch as a random effect and adjusted for autocorrelation between repeated measurements in time. The effect of introduced flow was determined by comparing differences between inflow and outflow of treatment ditches 1–4 to differences between inflow and outflow of control ditches 5–6. Effect of aquatic vegetation was determined by comparing differences between inflow and outflow of treatment ditches 2 and 3 that contained introduced aquatic vegetation to differences between inflow and outflow of treatment ditches 1 and 4 that did not contain introduced aquatic vegetation. Spearman correlations were performed to assess relationships between various sediment properties such as mean particle size, median particle size, OM, TP, pH, Soluble P, Fe/Al P, fulvic/humic P, Ca-Mg P, and residual P. Results were determined to be statistically significant when p < 0.05.

## Results

### 3.1 Sediment depth, organic matter, and pH

**Fig 1:**
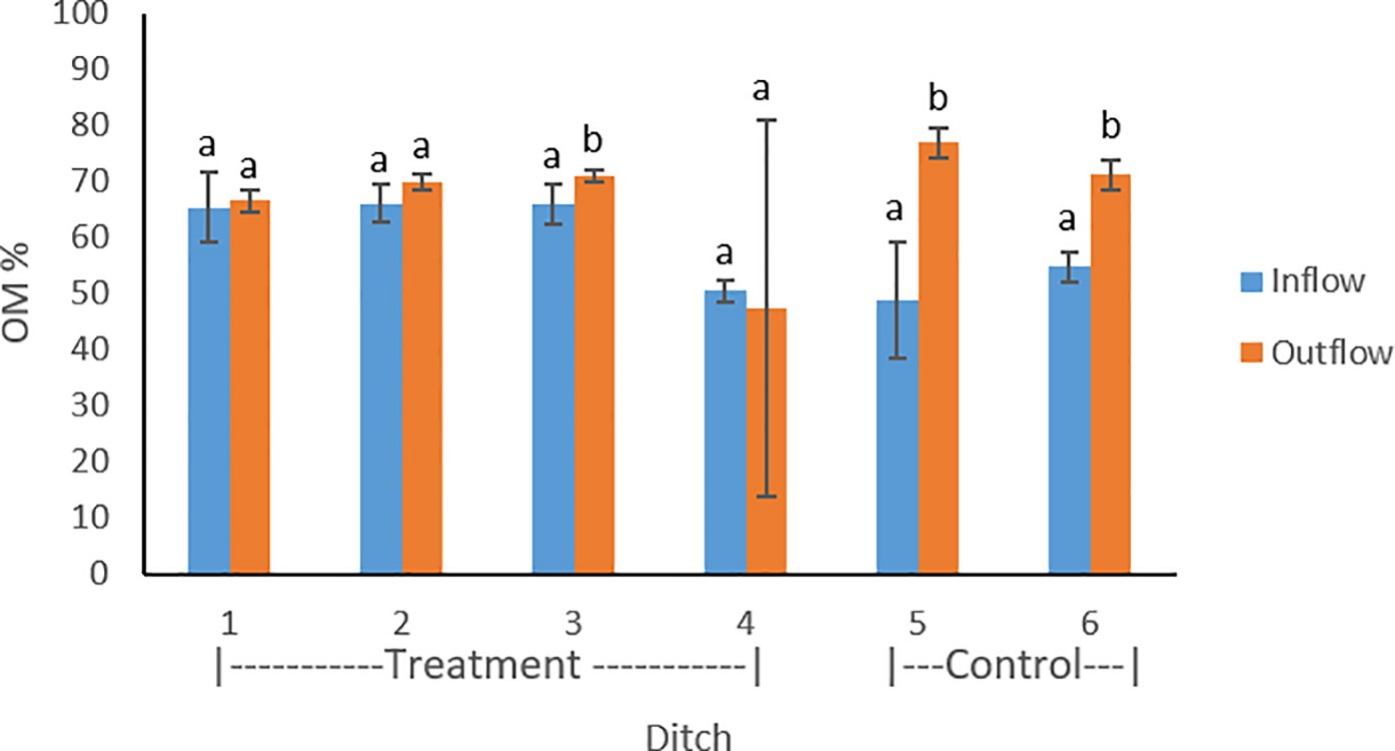
Organic matter measurement compared between inflow and outflow of the treatment (1–4) and control ditches (5–6). Error bars represent standard deviations throughout the three sampling periods.

**Fig 2:**
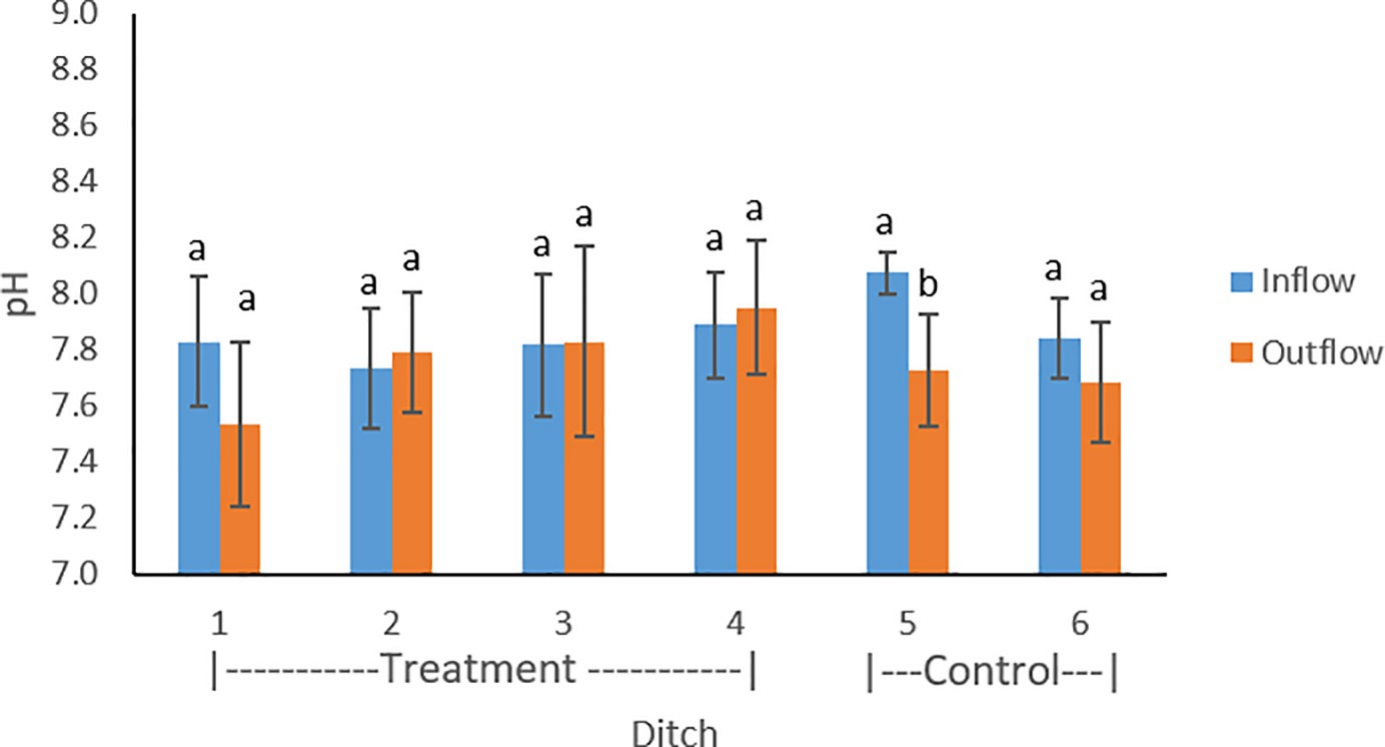
pH compared between inflow and outflow of the treatment (1–4) and control ditches (5–6). Error bars represent standard deviations throughout the three sampling periods.

Sediment depths ranged from 1.3–84cm, with an average value of 14 cm. Organic matter content of sediments within farm ditches ranged between 9.0 and 80%, with an average value of 63%. Lower average organic matter values were found in the inflow compared to outflow of treatment ditches 1–3 (Fig 1). Lower average organic matter was found in inflow sediments of control ditches compared to the outflow. Introduced flow (p<0.0001) and floating aquatic vegetation (p<0.0001) were found to have a significant effect on organic matter. Farm ditch sediment pH ranged between 7.3–8.2. On average, pH was slightly lower in the inflow compared to outflow locations of treatment ditches 2, 3, and 4 (Fig 2). Treatment ditch 1 and control ditches 5 and 6 contained lower average pH values in the outflow. Within the four treatment ditches, introduced flow (p = 0.33) and aquatic vegetation (p = 0.50) were not found to have a significant effect on pH.

### 3.2 Particle size

**Fig 3:**
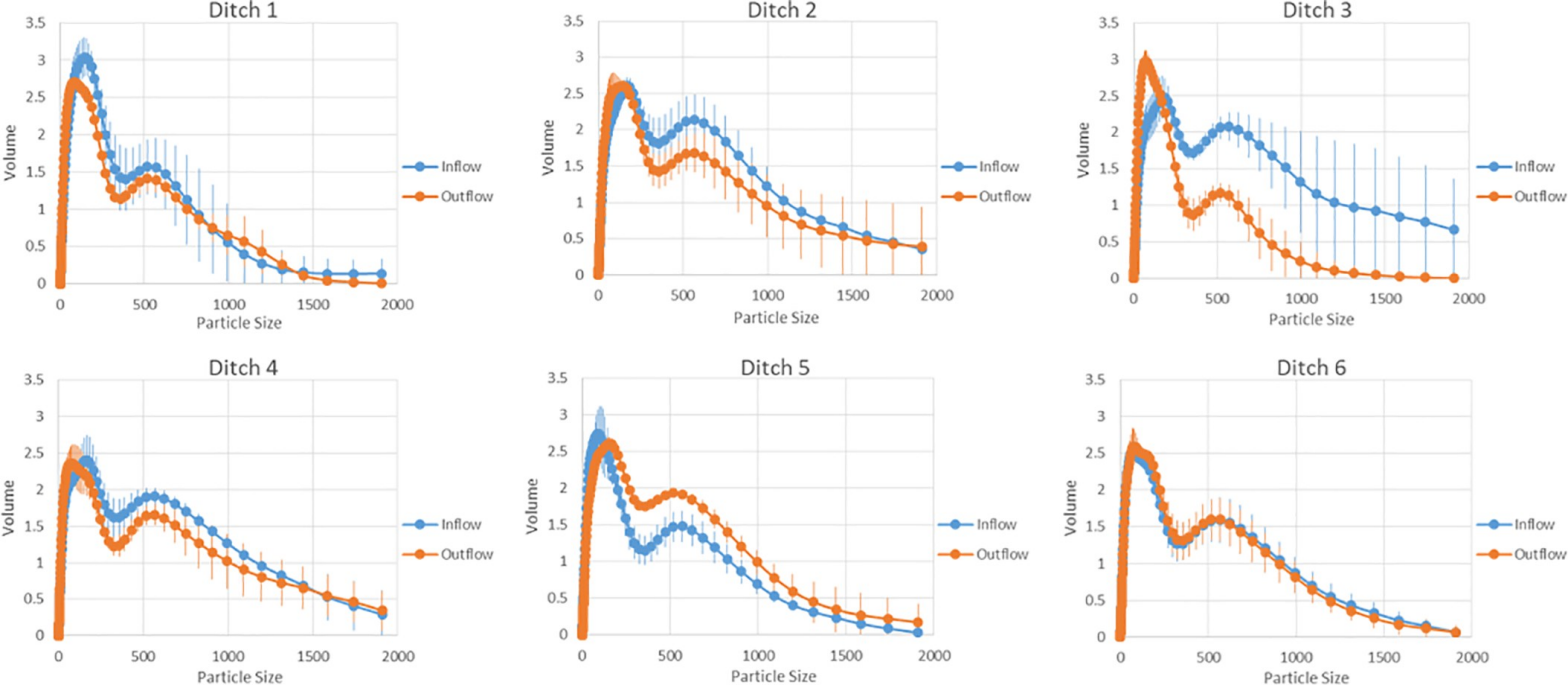
The average particle size distribution on a volumetric basis across the six field ditches. Treatment ditches (1–4) contain coarser average distribution in the inflow compared to the outflow. Control ditches (5–6) contain a finer average particle size distribution in the inflow compared to the outflow of control ditch 5. Control ditch 6 contains a similar average distribution in the inflow and outflow. Vertical blue and orange lines represent the standard deviation between three sampling periods for the inflow and outflow locations.

Average particle size distribution of the treatment ditches was coarser in inflow sediments compared to outflow (Fig 3). Control ditch 5 contained finer average particle size distribution in the inflow compared to the outflow. Control ditch 6 contained similar inflow and outflow average particle size distributions.

**Fig 4:**
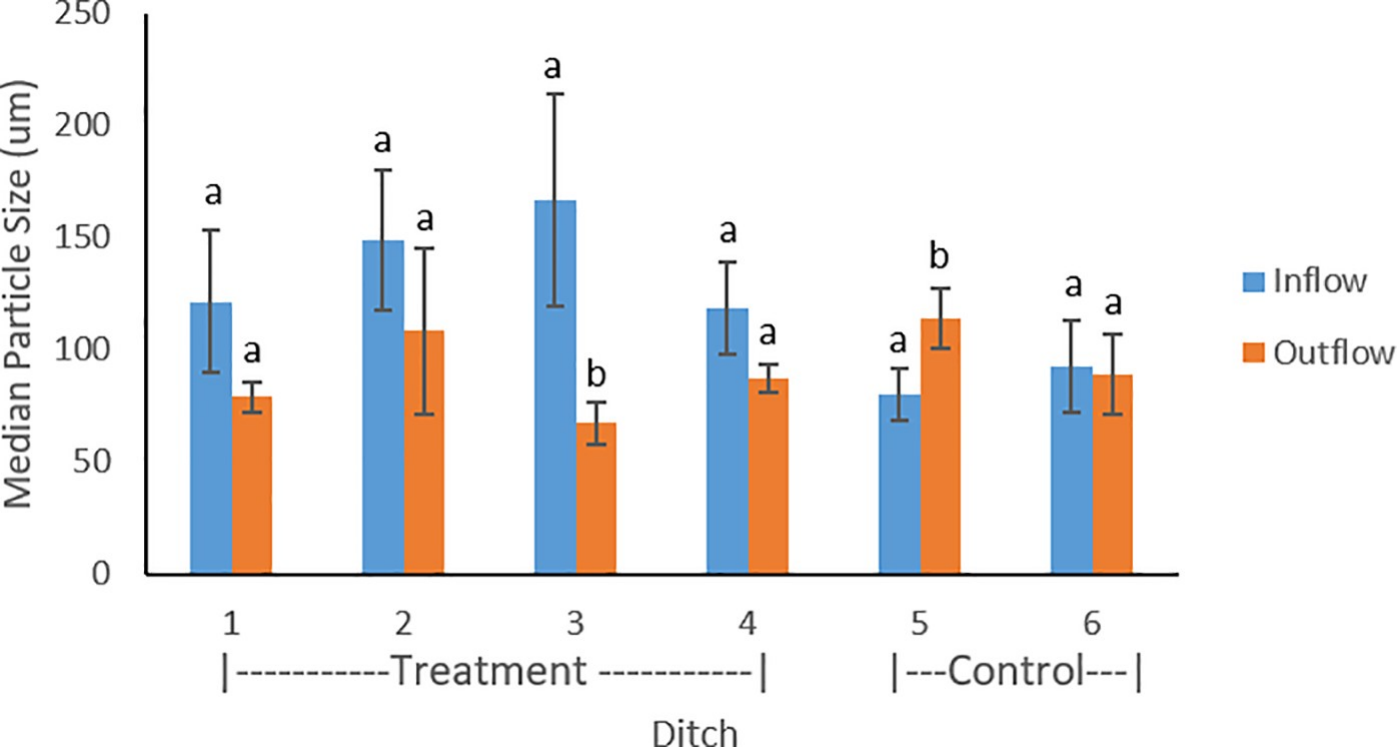
Median particle size compared between inflow and outflow of the treatment (1–4) and control ditches (5–6). Error bars represent standard deviations throughout the three sampling periods.

Median particle sizes ranged from 59.6–194 1μm in the USDA fine sand particle size class. For the November 2016 and 2017 sampling periods, each of the treatment ditches contained larger median particle size in the inflow compared to the outflow (Fig 4). In May 2017, smaller median particle sizes were found in the inflow of treatment ditches 1 and 2 compared to the outflow. On average there was larger median particle size recorded in the inflow of treatment ditches compared to outflow. Control ditch 5 contained finer particle size in the inflow compared to the outflow in each sampling period. Control ditch 6 contained similar average median particle sizes in the inflow and outflow. Within the four treatment ditches, introduced flow (p = 0.38) and floating aquatic vegetation (p = 0.43) were not found to have a significant effect on median particle size.

### 3.2 Total phosphorus

**Fig 5:**
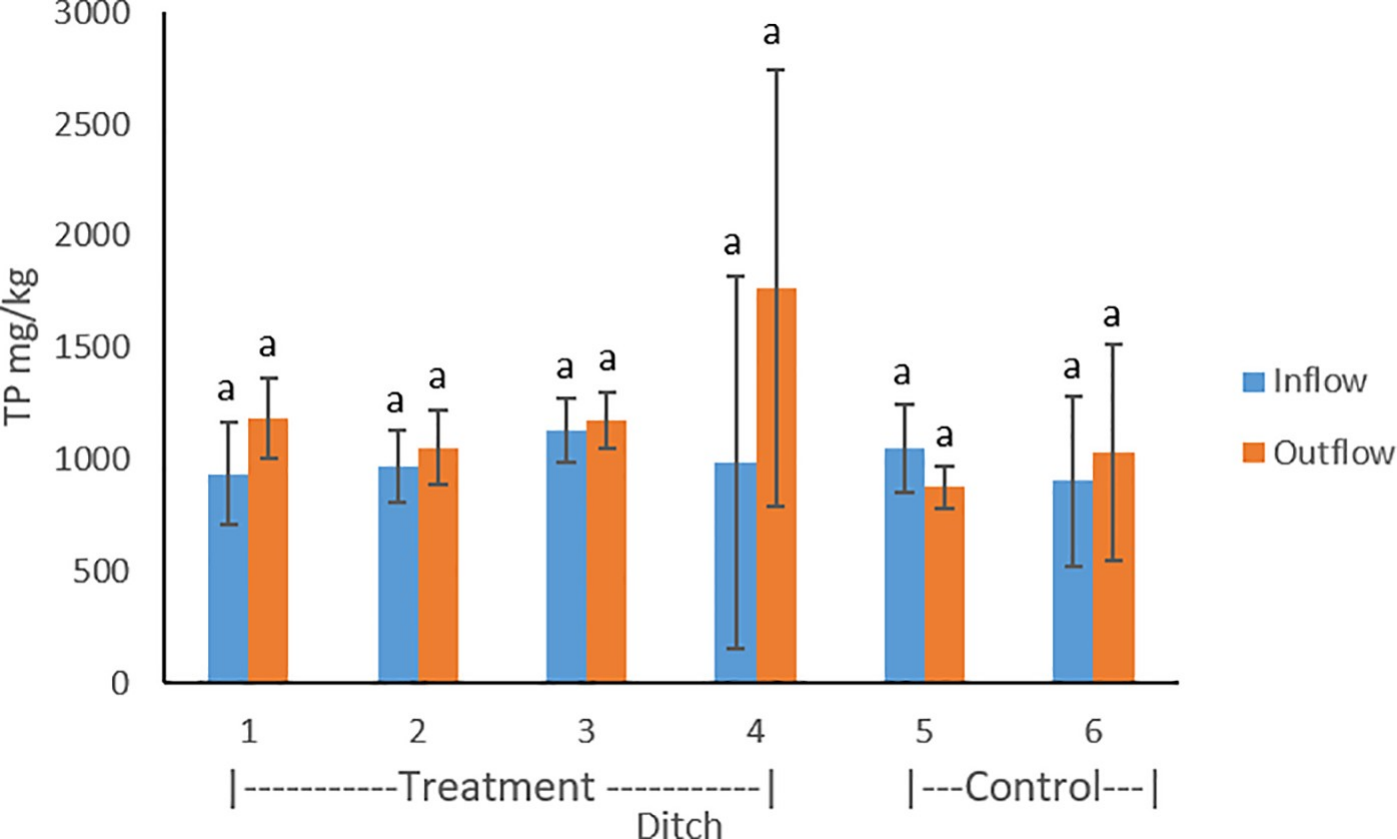
Sediment total phosphorus compared between inflow and outflow of the treatment (1–4) and control ditches (5–6). Error bars represent standard deviations throughout the three sampling periods.

Total P values ranged from 220–2889 mg kg^-1^, with an average value of 1086 mg kg^-1^. Lower average values of TP were found in inflow sediments of treatment ditches compared to the outflow (Fig 5). Control ditch 5 contained higher average TP in the inflow compared to the outflow, while control ditch 6 contained smaller average TP in the inflow sediments compared to the outflow. In May and November 2017 sampling periods, lower concentrations of TP were found in the inflow locations of the treatment ditches compared to the outflow. In November 2016, treatment ditches 1 and 3 contained lower TP concentrations in the inflow compared to the outflow, but the reverse trend occurred in treatment ditches 2 and 4. Control ditch 5 contained higher TP in the inflow compared to the outflow location in each of the sampling periods. Control ditch 6 contained lower TP in the November 2016 sampling period but higher TP in the two subsequent sampling periods. Within treatment ditches, introduced flow (p = 0.44) and floating aquatic vegetation (p = 0.54) were not found to have a significant effect on TP.

### 3.3 Phosphorus fractionation

**Fig 6:**
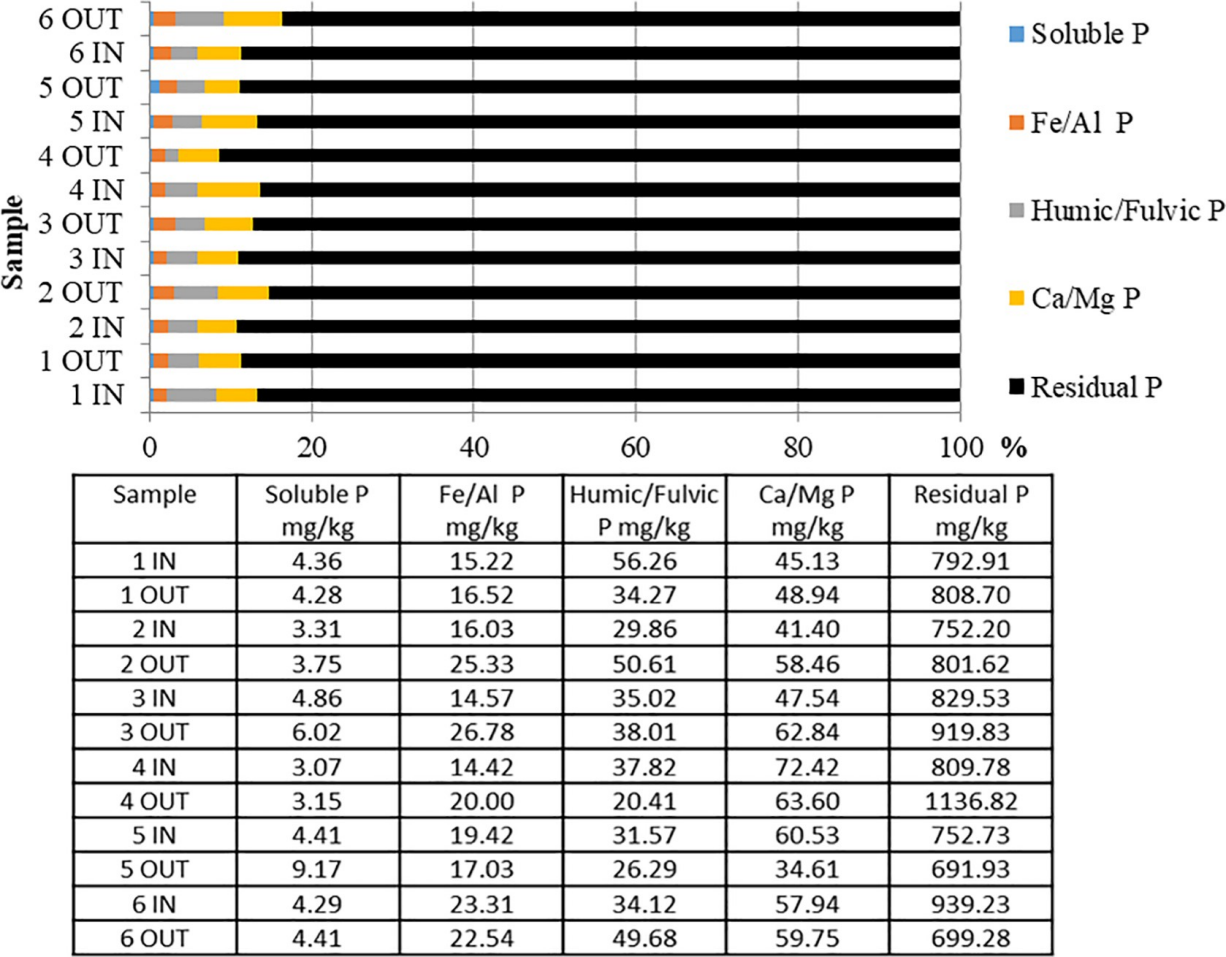
Results from modified Hedley (1982) phosphorus fractionation on a percentage basis (see horizontal bar chart above) and in mg kg^-1^.

The modified Hedley (1982) P fractionation recovered on average 97% of TP. The P recovered was on average 88% residual P, 5.8% Ca/Mg P, 3.3% fulvic/humic P, 2.1% Fe/Al, and 0.5% soluble P (Fig 6). Similar soluble P values were found in the inflow and outflow of treatment ditches 1, 2, and 4 and control ditch 6. Lower average values of soluble P were found in the inflow compared to the outflow locations of treatment ditch 3 and control ditch 6. Within treatment ditches, introduced flow (p = 0.09) and floating aquatic vegetation (p = 0.48) were not found to have a significant effect on soluble P. Treatment ditches contained, on average, lower Fe/Al P in the inflow compared to the outflow, while control ditches had higher Fe/Al P in inflow sediments compared to the outflow. Within treatment ditches, introduced flow (p = 0.39) and floating aquatic vegetation (p = 0.38) were not found to have a significant effect on Fe/Al P. The fulvic/humic P contained higher average P in the inflow locations compared to the outflow in treatment ditches 1, 3, and 4 and control ditch 5. Inflow locations contained lower values in treatment ditch 2 and control ditch 6. Within treatment ditches, introduced flow (p = 0.40) and floating aquatic vegetation (p = 0.67) were not found to significantly effect fulvic/humic P. The Ca/Mg P fraction contained lower average P in the inflow than outflow in treatment ditches 1–3 and control ditch 6. Treatment ditch 4 and control ditch 5 contained higher P values in inflow locations compared to the outflow. Introduced flow (p = 0.52) and floating aquatic vegetation (p = 0.59) were not found to have a significant effect on Ca/Mg P. Residual P contained lower P levels in the inflow compared to the outflow locations of the treatment ditches. The reverse trend was found in both control ditches. Within treatment ditches, introduced flow (p = 0.42) and aquatic vegetation (p = 0.75) were not found to have a significant effect on residual P.

### 3.4 P-31 NMR spectroscopy

**Fig 7:**
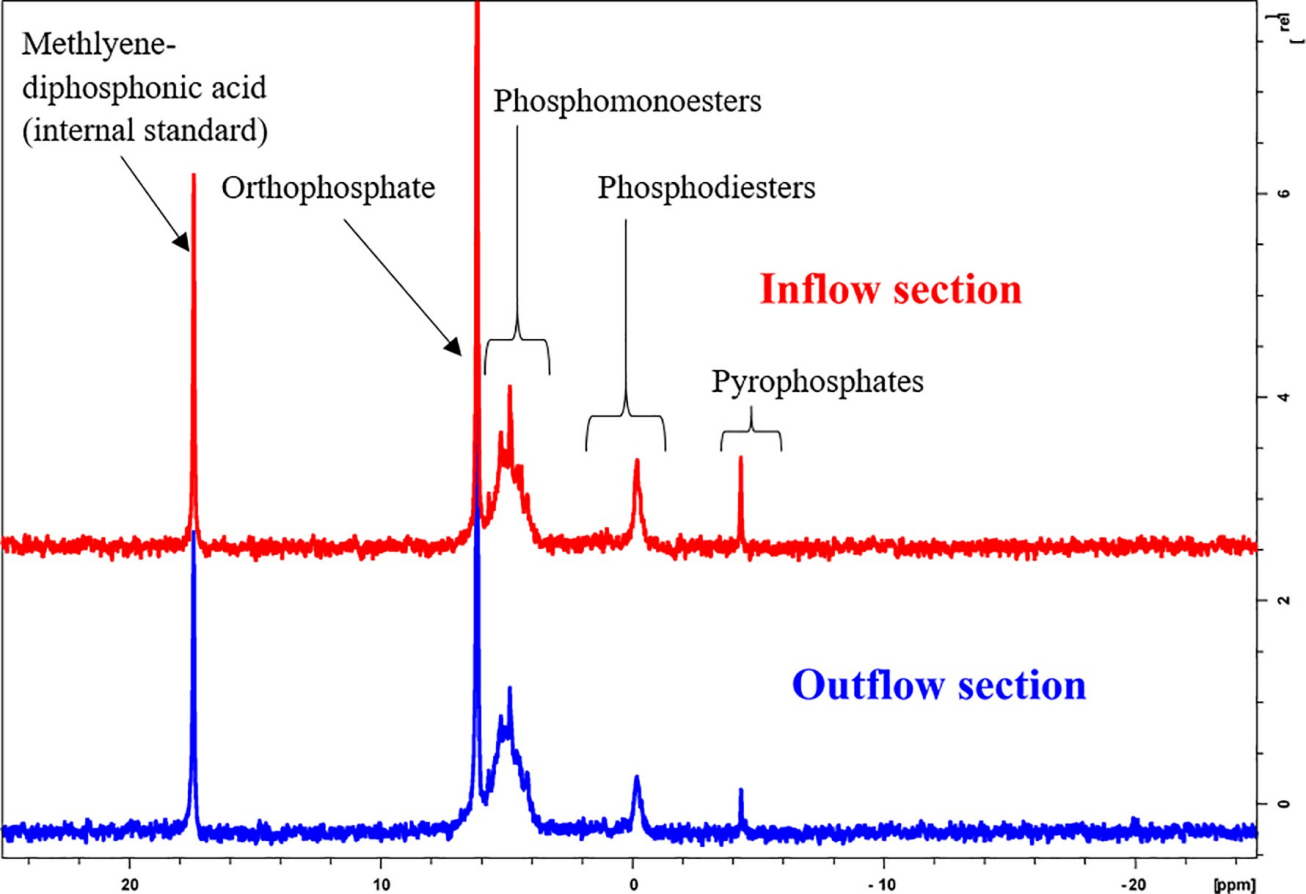
P-31NMR spectra of inflow and outflow sediments of treatment ditch 3.

Moisture content recovered by inflow sediment was 84% in the inflow and 78% in the outflow of treatment ditch 3. Samples examined for organic P by ^31^P NMR had slightly lower TP, NaOH EDTA TP, and 1 M HCl extracted Pi concentrations in the inflow compared to the outflow (Table 1). Of the organic forms of P recovered under the NaOH EDTA TP extraction, phosphomonoesters were the largest fraction followed by orthophosphate, phosphodiesters, and polyphosphates, respectively (Table 2). Phosphomonoesters and orthophosphates had lower concentrations in the inflow than the outflow, while phosphodiesters and polyphosphates contained higher concentrations in the inflow than the outflow (Fig 7). Higher Ca and Mn values were found in the inflow compared to the outflow. Iron values were lower in the inflow compared to the outflow.

**Table 1:** Sediment metal content. Characterization of inflow and outflow sediment samples from ditch 3.

Parameters	Unit	Inflow	Outflow
TP	mg kg^-1^	962.9	1006
Ca	g kg^-1^	80.3	55.8
Mn	mg kg^-1^	112.2	99.2
Fe	mg kg^-1^	7361	9020
HCl-P_i_	mg kg^-1^	469.5	482.5
NaOH-EDTA-TP	mg kg^-1^	537 ±6	567 ±13
HCl-Pi	(% of TP)	48.8	47.9
NaOH-EDTA-TP	(% of TP)	55.8	56.4

**Table 2:** Organic P forms (mg kg^-1^) of NaOH EDTA extractions in inflow and outflow samples collected from ditch 3.

Sample	NaOH-TP	Ortho Phosphate		Phospho Monoesters		Phospho diesters		Pyro phosphate	
	mg kg^-1^	mg kg^-1^	%	mg kg^-1^	%	mg kg^-1^	%	mg kg^-1^	%
Inflow	537	184.1	34.3	263.6	49.1	82.6	15.4	6.7	1.2
Outflow	567	206.2	36.4	293.3	51.7	61.8	10.9	5.7	1.0

### 3.5 Flow rates and TP

Flow rates in treatment ditches contained the same average flow at the inflow when pumps were operating. Mid ditch velocity rates differed by ditch depending on width and depth of the canal. Each treatment ditch had higher in average TP water concentration in the inflow compared to the outflow. Control ditch 5 and 6 had lower average TP water concentrations in the inflow compared to the outflow (Table 3).

**Table 3:** Practices used throughout experiment regarding flow rate, dredging, aquatic vegetation, and water total phosphorus (TP) concentrations.

Ditch No.	Flow Rate Inflow	Aquatic Vegetation	Water TP mg L^-1^
**1**	Hydraulic pumps provided flow of when operating 0.027 m^3^ s^−1^ Average mid ditch velocity rate of 0.047 m s^-1^	SAV introduced but not established. No FAV introduced.	0.089 mg L^-1^ Inflow 0.076 mg L^-1^ Outflow
**2**	Hydraulic pumps provided flow of when operating 0.027 m^3^ s^−1^ Average mid ditch velocity rate of 0.050 m s^-1^	SAV introduced but not established. FAV Water hyacinth introduced/established.	0.087 mg L^-1^ Inflow 0.075 mg L^-1^ Outflow
**3**	Hydraulic pumps provided flow of when operating 0.027 m^3^ s^−1^ Average mid ditch velocity rate of 0.053 m s^-1^	SAV introduced but not established. FAV Water lettuce introduced/established.	0.083 mg L^-1^ Inflow 0.080 mg L^-1^ Outflow
**4**	Hydraulic pumps provided flow of when operating 0.027 m^3^ s^−1^ Average mid ditch velocity rate of 0.039 m s^-1^	SAV introduced but not established. No FAV introduced.	0.086 mg L^-1^ Inflow 0.061 mg L^-1^ Outflow
**5**	No hydraulic pumps introduced	No FAV or SAV introduced.	0.091 mg L^-1^ Inflow 0.101 mg L^-1^ Outflow
**6**	No hydraulic pumps introduced	No FAV or SAV introduced.	0.098 mg L^-1^ Inflow 0.101 mg L^-1^ Outflow

All ditches dredged January of 2017 after first sediment sampling.

SAV = submerged aquatic vegetation

FAV = floating aquatic vegetation

### 3.6 Spearman correlation table

Median particle size was found to have a negative correlation with TP, but these correlations were not significant (p = 0.10) (Table 4). Negative correlation was found between median particle size and residual P but was not significant (p = 0.07). pH was found to have a negative significant correlation with fulvic and humic P. Correlations of particle size measurements mean and median were found to be significantly correlated (p<0.0001). Phosphorus indicators Total P, Fe/Al P, fulvic and humic P, Ca/Mg P, and residual P were also found to be significantly correlated with one another (p<0.05). Other P indicators, Fe/Al P and humic and fulvic P, contained negative significant correlations with one another, while Fe/Al P had a positive significant correlation to Ca/Mg P (p<0.05).

**Table 4:** Spearman correlation results. The top value represents the Spearman correlation coefficient, while the bottom number represents the corresponding P value.

Spearman Correlation Coefficients									
	Median	OM	TP	pH	Souble P	Fe/Al P	Humic/Fulvic P	Ca/Mg P	Residual P
**Median**	1	0.0540	-0.274	-0.1437	0.1142	-0.175	-0.0285	0.0350	-0.3050
	< .0001	0.7542	0.1047	0.4029	0.5069	0.3051	0.8686	0.8394	0.0705
**OM**		1	-0.2432	-0.1417	0.2417	0.2898	-0.0625	-0.074	-0.0764
		0.7542	0.1529	0.4097	0.1556	0.0864	0.7171	0.6642	0.6577
**TP**			1	0.0001	-0.039	-0.3353	0.5711	0.0332	0.4854
			0.1529	0.9994	0.8173	0.0455	0.0003	0.8475	0.0027
**pH**				1	0.0892	0.3010	-0.4678	0.2535	-0.0734
				0.9994	0.6046	0.0744	0.0040	0.1357	0.6704
**Soluble P**					1	0.0517	0.1531	0.2025	0.0391
					0.6046	0.7644	0.3725	0.2361	0.8208
**Fe/Al P**						1	-0.5204	0.4115	-0.1670
						0.7644	0.0011	0.0126	0.3302
**Humic/Fulvic P**							1	-0.0975	0.3510
							0.0011	0.5714	0.0358
**Ca/Mg P**								1	0.1086
								0.5714	0.5283
**Residual P**									1
									0.5283

## Discussion

Numerically, an average of 99.5% of the particles were clay. However, when particles were analyzed volumetrically, only 1.4% of the particles were clay. Larger particles such as pieces of inorganic limestone present in EAA histosols contain more volume, resulting in the discrepancy between numeric and volumetric results. The median was determined to be a better measurement of particle size compared to mean particle size due to the positive skew of the samples. The median measurement is less affected by the smaller number of mineral limerock particles that are larger in size and occupy more volume compared to organic particles that are smaller in size and occupy less volume (Table 5).

**Table 5:** Particle size classes based on USDA taxonomy displayed on both a volumetric and numeric basis.

	Nine USDA Particle Size Classes (Volumetric distribution %)									Three USDA Particle Size Classes		
	FClay	Clay	FSilt	CSilt	VFSand	FSand	MSand	CSand	VCSand	Clay	Silt	Sand
	<0.2 μm	0.2–2 μm	2–20 μm	20–50 μm	50–100 μm	100–250 μm	250–500 μm	500–1000 μm	1000–2000 μm	<2 μm	2–50 μm	50–2000 μm
1-IN	0.0	1.2	13.0	15.9	19.6	28.5	11.0	9.2	1.4	1.3	28.9	69.8
1-OUT	0.0	1.4	16.0	19.6	20.4	23.8	8.9	8.5	1.5	1.4	35.6	63.0
2-IN	0.0	1.1	11.5	14.1	16.1	24.5	13.5	14.5	4.7	1.2	25.6	73.2
2-OUT	0.0	1.2	13.2	16.6	18.5	24.5	10.8	11.3	3.9	1.2	29.8	69.0
3-IN	0.0	1.1	11.7	14.1	15.8	23.5	12.9	14.5	6.4	1.2	25.8	73.1
3-OUT	0.0	1.4	17.3	22.1	22.4	23.5	7.2	5.8	0.4	1.4	39.4	59.2
4-IN	0.1	1.5	13.7	15.9	16.0	22.6	12.0	13.5	4.8	1.6	29.6	68.9
4-OUT	0.0	1.5	16.1	18.5	17.9	20.9	9.5	11.3	4.4	1.5	34.5	64.0
5-IN	0.1	1.6	15.7	19.6	20.3	22.5	8.9	9.5	1.8	1.7	35.3	63.0
5-OUT	0.0	1.0	12.4	15.8	17.7	24.8	12.9	12.6	2.8	1.0	28.2	70.8
6-IN	0.0	1.6	15.5	19.8	20.1	22.4	8.9	9.4	2.3	1.6	35.4	63.0
6-OUT	0.0	1.4	14.2	16.1	19.0	23.6	11.2	11.9	2.5	1.4	30.4	68.2
	Nine USDA Particle Size Classes (Numeric distribution %)									Three USDA Particle Size Classes		
	FClay	Clay	FSilt	CSilt	VFSand	FSand	MSand	CSand	VCSand	Clay	Silt	Sand
	<0.2 μm	0.2–2 μm	2–20 μm	20–50 μm	50–100 μm	100–250 μm	250- 500 μm	500–1000 μm	1000–2000 μm	<2 μm	2–50 μm	50–2000 μm
1-IN	48.8	50.7	0.5	0.0	0.0	0.0	0.0	0.0	0.0	99.5	0.5	0.0
1-OUT	39.4	60.1	0.5	0.0	0.0	0.0	0.0	0.0	0.0	99.5	0.5	0.0
2-IN	38.0	61.5	0.6	0.0	0.0	0.0	0.0	0.0	0.0	99.4	0.6	0.0
2-OUT	30.0	69.3	0.7	0.0	0.0	0.0	0.0	0.0	0.0	99.3	0.7	0.0
3-IN	39.5	59.9	0.6	0.0	0.0	0.0	0.0	0.0	0.0	99.4	0.6	0.0
3-OUT	33.2	66.1	0.7	0.0	0.0	0.0	0.0	0.0	0.0	99.3	0.7	0.0
4-IN	67.9	31.8	0.3	0.0	0.0	0.0	0.0	0.0	0.0	99.7	0.3	0.0
4-OUT	42.3	57.1	0.6	0.0	0.0	0.0	0.0	0.0	0.0	99.4	0.6	0.0
5-IN	58.2	41.4	0.4	0.0	0.0	0.0	0.0	0.0	0.0	99.6	0.4	0.0
5-OUT	43.9	55.6	0.5	0.0	0.0	0.0	0.0	0.0	0.0	99.5	0.5	0.0
6-IN	55.7	43.9	0.4	0.0	0.0	0.0	0.0	0.0	0.0	99.6	0.4	0.0
6-OUT	24.0	75.2	0.9	0.0	0.0	0.0	0.0	0.0	0.0	99.1	0.9	0.0

F = fine, C = coarse, M = medium, VC = very coarse

Introduced flow was not found to cause significant differences in particle size between inflow and outflow sediments in treatment ditches. However, apart from the May 2017 sampling, there was a general trend in treatment ditches of coarser textured sediments located in the inflow compared to the outflow. This may be due to increased mineral content in the inflow sediments, which generally contained slightly lower organic matter. Calcium content in sediments sampled for treatment ditch 3 was determined to be higher in the inflow sediment (80308 mg kg^-1^) than the outflow sediment (55868 mg kg^-1^) (Table 1). Introduced flow may cause coarser particles to settle near the inflow and finer particles to settle near the outflow. Finer particles already present in ditch sediment may also be pushed to the back of the ditch overtime. Sediments were dredged by the farmer in January 2017 as a best management practice to reduce P runoff, which could have affected the results in the subsequent May 2017 sampling. The rainy season in south Florida begins in May and ends in November. Over time, rainfall could influence particle size in ditch sediments by increasing the erosion of organic particulates from adjacent sugarcane fields.

Although TP had on average an inverse trend compared to particle size, the two parameters were not significantly correlated. Lower average sediment TP concentrations were found in the inflow compared to the outflow. In contrast, higher TP water concentrations were found in the inflow compared to the outflow. It is unclear how the reduction of TP concentrations in water flowing between inflow and outflow affected sediment TP concentrations. Median particle size did not contain a significant negative correlation with any of the fractions of P recovered in the modified Hedley (1982) fractionation procedure. The residual P fraction contained most of the P recovered and was closest to containing a significant negative correlation with median particle size (p = 0.07). It is surprising that TP was negatively correlated with organic matter because TP is often positively correlated with organic matter [32,33]. Although the correlation (p = 0.15) was not significant, the negative correlation may indicate that much of the P present is in the inorganic rather than organic form. In this region, sediments have been found to contain a large fraction of P in the inorganic Ca/Mg P form due to the presence of limerock [21,34]. Phosphorus recovered in the modified Hedley (1982) P fractionation was lower in Ca/Mg P and higher in residual P compared to previous studies on soils and canal sediments in this region [21,26]. Ditch sediments are generally shallower and likely contain more consolidated limestone compared to canal sediment because canals are dug to a deeper depth disturbing the underlying limerock [21]. Past studies have found organic sediments to have low ability to remove P from the water column [35,36]. Calcareous clay loams have a high removal capacity and are similar to the underlying limestone deposits in the EAA [36].

There was no trend in soluble P between inflow and outflow sediments of treatment ditches. For the more labile organic P fractions recovered using ^31^P NMR spectroscopy, higher concentrations of labile and moderately labile P species pyrophosphates and phosphodiesters were discovered in the inflow compared to the outflow sediment. Phosphomoesters are generally considered a more recalcitrant form of P and were slightly lower in the inflow location compared to the outflow. Phosphodiesters are comparatively poorly stabilized in the extracellular environment, leading to a greater lability and potential for biological turnover. Larger values of orthophosphate and labile organic P were recovered using ^31^ P NMR spectroscopy compared to the modified Hedley (1982) P fractionation. Phosphorus-31 NMR spectroscopy is considered an advanced method of P fractionation [37]. Therefore, the modified Hedley (1982) P fractionation may be underestimating labile fractions of P and overestimating the residual P fraction. Approximately 50% of the organic P recovered using ^31^P NMR spectroscopy was found to be in the more recalcitrant phosphomonoester form, while in the modified Hedley (1982) 88.29% was recovered as residual P.

The influence of flow on particle size does not appear to be large enough to have a significant impact on labile P or TP. Flow rates provided to each of the four treatment ditches were relatively low (0.027 m^3^ s^−1^) and only operated on a 10:10 min on:off schedule from 8:00am– 6:00pm. Since these sediments are primarily organic, this paper hypothesized that most of the P would be in the organic form and smaller average particle size would correlate with TP. Phosphorus-31 NMR spectroscopy results showed that 44.18% and 43.64% of TP was in the inorganic form in the inflow and outflow of treatment ditch 3. A large component of TP is in the inorganic form and the inorganic limerock in the ditches can be visibly observed to contain larger particle size compared to the organic particles. There was a general trend of lower P content in the inflow sediments that contained larger average particle size compared to outflow sediments of the treatment ditches.

Introduced flow and aquatic vegetation within treatment ditches were only found to have a significant effect on organic matter. Control ditches contained significantly larger differences in organic matter between inflow and outflow compared to treatment ditches. Lower levels of organic matter were found in the inflow compared to the outflow of the control ditches. The percent cover of floating aquatic vegetation did not appear to decrease until after the November 2017 sediment sampling. The presence of aquatic vegetation is not expected to have an impact on particle size or P-dynamics in ditch sediments. Reduction in water TP concentrations could partially be due to the uptake of nutrients by aquatic vegetation, especially in treatment ditches 2 and 3, where water hyacinth and water lettuce were introduced, respectively. If ditches are continued to be managed with aquatic vegetation and without dredging the sediments in these ditches are expected to become more organic and flocculent over time, reducing their ability to adsorb P [36].

## Conclusions

On average inverse trends between sediment particle size and TP appeared between the inflow and outflow. In the sediment, lower P concentrations were found in the inflow compared to the outflow, and larger particle size in the inflow compared to the outflow. However, introduced flow and aquatic vegetation were not found to have a significant effect on particle size or TP. Particle size and TP were not significantly correlated. The presence of limerock likely contains higher average particle size and increased P adsorption capacity compared to the organic fraction of the sediments. Water TP concentrations were found to decrease between the inflow and outflow of the treatment ditches due to the immobilization of P and through biogeochemical processes that reduce P content in ditch water. Large discrepancies were found between particle size analysis on volumetric and a numerical basis. Future studies using laser diffraction particle size analysis on organic soils and sediments should consider analyzing results on both a volumetric and numeric basis to determine how results should be presented for a given sample. Laser diffraction particle size analysis and ^31^P NMR spectroscopy offer analytical techniques to further our understanding of the relationship between particle size and P dynamics.

## Supporting information

S1 Dataset Supplemental data. (XLSX)

## References

[1] Huang L, Li L, Huang L, Gielen G, Zhang Y, Wang H. Influence of incubation time on phosphorus sorption dynamics in lake sediments. Journal of Soils and Sediments. 2012; 12: 443–455

[2] Childers DL, Doren R, Jones R, Noe GB, Rugge M, Scinto LJ. Decadal change in vegetation and soil phosphorus pattern across the Everglades landscape. Journal of Environmental Quality. 2003; 32: 344–362 10.2134/jeq2003.3440 12549575

[3] Gunderson LH. Vegetation of the Everglades: Determinants of community composition In Davis S. M. and Ogden J. C. (eds), Everglades: The Ecosystem and its Restoration. St. Lucie Press, Delray Beach (FL) 1994; 323–340

[4] Rice RW, Bhadha J, Lang T, Daroub S, Baucum L. Farm-level phosphorus-reduction best management practices in the Everglades Agricultural Area. In Proceedings of the Florida State Horticultural Society. 2013; 126: 300–304

[5] Rice RW, Gilbert RA, Daroub SH. Application of the soil taxonomy key to the organic soils of the Everglades Agricultural Area. 2005; University of Florida document SS-AGR-246

[6] Snyder GH. Everglades Agricultural Area soil subsidence and land use projections. 2005. In Proceedings

[7] Snyder GH. Soils of the EAA. Everglades Agricultural Area (EAA): Water, Soil, Crop, and Environmental Management, 1994; 3: 27–41

[8] USDA subsidence study of the Everglades Agricultural Area. Soil Conservation Service Greenacres Field Office 1988 Greenacres, Fl

[9] Daroub SH, Lang TA. Annual report submitted to Florida Department of Environmental Protection. Everglades Research and Education Center. Institute of Food and Agricultural Sciences. University of Florida. 2016.

[10] Daroub SH, Stuck JD, Lang TA, Diaz OA. Particulate phosphorus in the everglades agricultural area: I–Introduction and sources. Soil Water Department of University of Florida IFAS Extension Publication SL. 2002; 197

[11] Bhadha JH, Lang T A, Gomez SM, Daroub SH, Giurcanu MC. Effect of water lettuce and filamentous algae on phosphorus loads in farm canals in the Everglades Agricultural Area. Journal of Aquatic Plant Management. 2015; 53: 44–53

[12] Brix H. Do macrophytes play a role in constructed treatment wetlands? Water Science and Technology. 1997; 35: 11–17

[13] Zhu Y, Zhang R, Wu F, Qu X, Xie F, Fu Z. Phosphorus fractions and bioavailability in relation to particle size characteristics in sediments from Lake Hongfeng, Southwest China. Environmental Earth Sciences. 2013; 68: 1041–1052

[14] Suñer L, Galantini JA. Texture influence on soil phosphorus content and distribution in semiarid pampean grasslands. International Journal of Plant Science. 2015; 7: 109–120

[15] Bhadha JH, Harris WG, Jawitz JW. Soil phosphorus release and storage capacity from an impacted subtropical wetland. Soil Science Society of America Journal. 2010; 74:1816–1825

[16] Cole CV, Olsen SR. Phosphorus solubility in calcareous soils: II. Effects of exchangeable phosphorus and soil texture on phosphorus solubility. Soil Science Society of America Journal. 1959; 23: 119–121

[17] Zheng Z, Parent LE, MacLeod JA. Influence of soil texture on fertilizer and soil phosphorus transformations in Gleysolic soils. Canadian Journal of Soil Science. 2003; 83: 395–403

[18] Dong A, Simsiman GV, Chesters G. Particle-size distribution and phosphorus levels in soil, sediment, and urban dust and dirt samples from the Menomonee River Watershed, Wisconsin, USA. Water Research. 1983; 17: 569–577

[19] Huffman SA, Cole CV, Scott NA. Soil texture and residue addition effects on soil phosphorus transformations. Soil Science Society of America Journal. 1996; 60: 1095–1101

[20] O’Halloran IP, Kachanoski RG, Stewart JWB. Spatial variability of soil phosphorus as influenced by soil texture and management. Canadian Journal of Soil Science. 1985; 65: 475–487

[21] Das J, Daroub SH, Bhadha JH, Lang TA, Diaz O, Harris W. Physicochemical assessment and phosphorus storage of canal sediments within the Everglades Agricultural Area, Florida. Journal of Soils and Sediments. 2012; 12: 952–965

[22] Villapando RR, and Graetz DA. Phosphorus sorption and desorption properties of the spodic horizon from selected Florida Spodosols. Soil Science Society of America Journal. 2001; 65: 331–339

[23] Hedley MJ, Stewart JW. Method to measure microbial phosphate in soils. Soil Biology and Biochemistry. 1982; 14: 377–385

[24] Reddy KR, Wang Y, DeBusk WF, Fisher MM, Newman S. Forms of soil phosphorus in selected hydrologic units of the Florida Everglades. Soil Science Society of America Journal. 1998; 62: 1134–1147

[25] Eshel G, Levy GJ, Mingelgrin U, Singer MJ. Critical evaluation of the use of laser diffraction for particle-size distribution analysis. Soil Science Society of America Journal. 2004; 68: 736–743

[26] Zobeck TM. Rapid soil particle size analyses using laser diffraction. Applied Engineering in Agriculture. 2004; 20: 633

[27] United States Environmental Protection Agency. Method 365.1, Revision 2.0: Determination of Phosphorus by Colorimetry. 1993; Available from: https://www.epa.gov/sites/production/files/2015-08/documents/method_365-1_1993.pdf

[28] Cade-Menun BJ, Preston CM. A comparison of soil extraction procedures for ^31^P NMR spectroscopy. Soil Science. 1996; 161, 770–785

[29] Tuner BL, Mahieu N, Condron LM. Phosphorus-31 nuclear magnetic resonance spectral assignments of phosphorus compounds in soil NaOH–EDTA extracts. Soil Science Society of AmericaJournal. 2003; 67: 497–510

[30] Turner BL, Cade-Menun B, Condron LM, Newman S. Extraction of soil organic phosphorus. Talanta. 2005; 66: 294–306 10.1016/j.talanta.2004.11.012 18969994

[31] Bhadha JH, Lang TA, Gomez SM, Daroub SH, Giurcanu MC. Effect of aquatic vegetation on phosphorus loads in the Everglades Agricultural Area. Journal of Aquatic Plant Management. 2015; 53: 44–53

[32] House WA, Denison FH. Total phosphorus content of river sediments in relationship to calcium, iron and organic matter concentrations. Science of the Total Environment. 2002; 282: 341–351 10.1016/s0048-9697(01)00923-8 11846078

[33] Dalai R. C. Soil organic phosphorus. Advances in Agronomy. 1977; 29: 83–117.

[34] Bhadha JH, Lang TA, Daroub SH. Seasonal delivery of organic matter and metals to farm canals: Effect on sediment phosphorus storage capacity. Journal of Soils and Sediments. 2014; 14: 991–1003

[35] Reddy KR. Soluble phosphorus release from organic soils. Agriculture, Ecosystems and Environment. 1983; 9: 373–382

[36] Das J, Daroub SH, Bhadha JH, Lang TA, Josan M. Phosphorus release and equilibrium dynamics of canal sediments within the Everglades Agricultural Area, Florida. Water, Air, and Soil Pollution. 2012; 223: 2865–2879

[37] Negassa W, Leinweber P. How does the Hedley sequential phosphorus fractionation reflect impacts of land use and management on soil phosphorus: A review. Journal of Plant Nutrition and Soil Science. 2009; 172: 305–325

